# Synergism between the Antidepressant Sertraline and Caspofungin as an Approach to Minimise the Virulence and Resistance in the Dermatophyte *Trichophyton rubrum*

**DOI:** 10.3390/jof8080815

**Published:** 2022-08-03

**Authors:** Carlos H. Lopes Rocha, Flaviane M. Galvão Rocha, Tamires A. Bitencourt, Maíra P. Martins, Pablo R. Sanches, Antonio Rossi, Nilce M. Martinez-Rossi

**Affiliations:** Department of Genetics, Ribeirão Preto Medical School, University of São Paulo, USP, Ribeirao Preto 14049-900, SP, Brazil; henriqueclr16@usp.br (C.H.L.R.); flavianerocha@usp.br (F.M.G.R.); tabitencourt@yahoo.com.br (T.A.B.); mairapompeu@hotmail.com (M.P.M.); psanches@usp.br (P.R.S.); anrossi@usp.br (A.R.)

**Keywords:** *Trichophyton rubrum*, dermatophyte, synergistic combinations, antifungal resistance, sertraline, caspofungin and biofilm

## Abstract

*Trichophyton rubrum* is responsible for several superficial human mycoses. Novel strategies aimed at controlling this pathogen are being investigated. The objective of this study was to evaluate the antifungal activity of the antidepressant sertraline (SRT), either alone or in combination with caspofungin (CASP). We calculated the minimum inhibitory concentrations of SRT and CASP against *T. rubrum*. Interactions between SRT and CASP were evaluated using a broth microdilution chequerboard. We assessed the differential expression of *T. rubrum* cultivated in the presence of SRT or combinations of SRT and CASP. We used MTT and violet crystal assays to compare the effect of SRT alone on *T. rubrum* biofilms with that of the synergistic combination of SRT and CASP. A human nail infection assay was performed. SRT alone, or in combination with CASP, exhibited antifungal activity against *T. rubrum.* SRT targets genes involved in the biosyntheses of cell wall and ergosterol. Furthermore, the metabolic activity of the *T. rubrum* biofilm and its biomass were affected by SRT and the combination of SRT and CASP. SRT alone, or in combination, shows potential as an approach to minimise resistance and reduce virulence.

## 1. Introduction

Dermatophytosis is a common fungal infection caused by dermatophytes, a class of non-opportunistic pathogens that obtain nutrients from keratinised tissues such as hair, skin, and nails. *Trichophyton rubrum* is a cosmopolitan species often isolated from cutaneous infections and immunocompromised human hosts worldwide, resulting in severe conditions that may lead to public health issues [1]. It is estimates that dermatophytes affect approximately 25% of the world population, wherein 30–70% of adults act as carriers who do not present with clinical manifestations [2]. The damp climate and elevated temperatures of tropical and subtropical regions contribute to the high rate of dermatophytosis [1].

Treating *T. rubrum* infections is challenging because only a few therapeutic options, such as azoles and allylamines that interfere with the ergosterol biosynthesis pathway, are available. Furthermore, in addition to lengthy and costly treatments, several antifungal resistance cases have been reported [3]. Primary defence mechanisms of the host include skin peeling, decreased humidity, skin pH, elevated temperature, and fatty acids. In contrast, fungi develop adaptive responses to overcome these challenges [4]. Fungi trigger several mechanisms that overexpress drug efflux pumps, detoxify enzymes, and modify drug targets, all of which are aimed at tolerating or resisting the effects of antifungals [3,5].

Additionally, surface-based cell population complexes, termed biofilms, contribute to drug resistance, mainly by resisting penetration. These complex structures manifest in the form of an extracellular matrix, characterised by metabolic heterogeneity and upregulated efflux pump-related genes [6,7]; thus, the tracking and identification of compounds with antifungal activity are urgently required [8,9]. In this regard, drug repositioning appears to be an exciting alternative. When repurposing an existing drug for a newer use, drug characteristics, such as toxicity and pharmacokinetics, must already be established [10].

Combining sertraline (SRT), one of the most prescribed antidepressants explored for its antifungal properties, with commercial antifungals represents a promising therapeutic approach that would enhance the therapeutic efficacy [11,12]. SRT significantly reduced the pulmonary fungal burden associated with *Aspergillus fumigatus* infection in a murine aspergillosis model. The same study reported that SRT ensured the survival of 25% of infected larvae of a *Galleria mellonella* aspergillosis model, compared to the high mortality rates observed in infected and untreated larvae [13].

In mammals, SRT selectively inhibits serotonin reuptake by locking the 5-hydroxytryptamine (5-HT) transporter [14]. In yeast cells, SRT targets the phospholipid membranes of acidic organelles, which are part of an intracellular vesicle transport system [15]. SRT also exerts antifungal effects by disrupting translation, thereby inhibiting protein synthesis [11].

A synergistic combination of SRT and amphotericin B improves inhibitory activity against *A. fumigatus*. Notably, combining SRT with itraconazole was also synergistic [16]. Essays on *Cryptococcus neoformans* evidenced the antifungal activity of SRT, either in monotherapy or in synergic combination with fluconazole [16,17]. In addition, SRT exhibited synergistic effects against *Trichosporon asahii* planktonic cells with caspofungin (CASP) [18]. 

The activity of CASP against dermatophytes is not well defined. Pioneering works have demonstrated that CASP shows excellent activity against the dermatophytes *T. rubrum, Trichophyton interdigitale,* and *Microscoporum canis* [19,20]. Another study showed that CASP promotes several morphological changes, including shortening and induction of aberrant hyphae growth; however, it has reduced efficacy and incompletely inhibited in vitro growth [21].

There are few studies demonstrating the CASP effect with other antifungal agents. In most cases, the combination therapy of CASP with another agent is used for treating infections caused by the genus *Candida* [22,23]. In association with farnesol, CASP significantly inhibited the metabolic activity of *Candida parapsilosis* cells [24]. CASP, with fluconazole and posaconazole, is an effective strategy against *Candida glabrata* [22]. CASP belongs to a class of antifungal agents known as echinocandins, which inhibit the synthesis of the fungal cell wall component, beta-(1,3)-D-glucan.

Thus, owing to the necessity of expanding the range of therapeutics available against dermatophytosis, we hypothesised that SRT combined with CASP might help minimise drug resistance. Furthermore, we evaluated the expression of genes associated with the resistance of *T. rubrum* to SRT alone and its combination with CASP.

## 2. Materials and Methods

### 2.1. Strain and Growth Conditions

The *T. rubrum* strain, CBS118892 (Westerdijk Fungal Biodiversity Institute, Utrecht, Netherlands), obtained from a patient with onychomycosis, was cultivated on malt extract agar (Becton Dickinson, Franklin Lakes, NJ, USA) for 35 days at 28 °C, as previously described [25], for total RNA extraction. The fungal suspension was prepared in 0.9% NaCl, following which the conidia concentration was estimated using a Newbauer chamber. Approximately 1 × 10^6^ conidia were added to 100 mL of liquid Sabouraud (SB) at pH  5.7 supplemented with 2% glucose and 1% peptone (Becton Dickinson, Franklin Lakes, NJ, USA), followed by incubation at 28 °C for 96 h under continuous shaking. The resulting mycelia were then transferred to 100 mL of SB in the presence of a sublethal dose (70 mg/L) of SRT (Cayman Chemical, Ann Arbor, MI, USA), in the presence of a sublethal combination (sc) of 0.273 mg/L SRT + 10.93 mg/L CASP (Merck Sharp & Dohme, São Paulo, SP, Brazil), 0.273 mg/L SRT, 10.93 mg/L CASP, and in the absence of drugs (control), followed by incubation at 28 °C with shaking (120 rpm) for 3 h and 12 h.

### 2.2. Minimum Inhibitory Concentration (MIC) and Interaction between SRT and CASP

MICs were obtained according to the M38-A reference method recommended by the Clinical and Laboratory Standards Institute (CLSI) [26], with the following modification: 100 μL of the conidial suspension, amounting to 6 × 10^4^ conidia/mL, was added to each well in the 96-well microtitre plate. The final conidial concentration was adjusted to approximately 3 × 10^4^ conidia per well in RPMI 1640 (Sigma-Aldrich, St Louis, MO, USA) or SB medium. RPMI was buffered with 0.165 M morpholinepropanesulfonic acid (MOPS) (Sigma-Aldrich), and the pH was adjusted to 7.0. SRT and CASP were prepared as stock solutions in dimethyl sulfoxide (DMSO, Sigma-Aldrich): SRT (50,000 mg/L); and CASP (5000 mg/L). Serial dilutions of SRT and CASP were performed in RPMI medium, buffered with 0.165 M morpholinepropanesulfonic acid (MOPS), or SB medium. The final concentrations of SRT ranged between 0.78–200 mg/L, whereas those of CASP ranged between 0.98–250 mg/L. Interactions between SRT and CASP were evaluated using a broth microdilution chequerboard and quantified using the fractional inhibitory concentration index (FICI): FICI ≤ 0.5, FICI > 0.5 to ≤ 4.0, and FICI > 4.0 were categorised as synergism, indifference, and antagonism, respectively [27]. FICIs were calculated for all possible combinations of different concentrations. The results were expressed as the mean of FICIs. Assay plates were used to determine MICs, and the broth microdilution chequerboard was incubated at 28 °C for seven days. Growth controls were performed in wells containing only the fungal suspension and medium. Individual and combined MICs were defined by comparison with growth controls performed in wells containing only fungal suspension and media, with complete growth inhibition. All experiments were performed in triplicate.

### 2.3. Total RNA Extraction and cDNA Synthesis

Total RNA was extracted from *T. rubrum*, cultivated in the presence of SRT alone and SRT combined with CASP, using an Illustra RNAspin mini-isolation kit (GE Healthcare, Chicago, IL, USA). Mycelia were ground via mechanical pulverisation using a mortar and pestle in liquid nitrogen, and RNA samples were treated with RNase-free DNase I (Sigma-Aldrich). Complementary DNA (cDNA) was synthesised from each condition containing 1000 ng of total RNA in a 20 μL reaction volume using a High-Capacity cDNA Synthesis kit (Applied Biosystems, Waltham, MA, USA). Equal amounts of RNA from three independent biological replicates were used to synthesise cDNA.

### 2.4. RT-qPCR Analysis

Gene expression was quantified via qPCR using a StepOnePlus Real-Time PCR system (Applied Biosystems). PCR reactions were performed with specific primer pairs designed using Prime3Plus software (https://www.bioinformatics.nl/cgi-bin/primer3plus/primer3plus.cgi (accessed on 4 July 2022)) and specificity (Table S1). Each qPCR reaction was performed using a final volume of 12.5 μL: 0.5 μL primer, 6.25 μL SYBR Green PCR Master Mix (Applied Biosystem, Waltham, MA, USA), and 70 ng cDNA. The thermocycler conditions for RT-qPCR were 95 °C for 10 min, followed by 40 cycles of 95 °C for 15 s and 60 °C for 1 min. We selected genes encoding the enzymes glyceraldehyde 3 phosphate dehydrogenase (*gapdh*) and DNA-dependent RNA polymerase II (*rpb2*) as endogenous controls. Data were derived from three independent replicates, and the 2^−∆∆ct^ method was used to assess the relative quantification of responsive genes [28].

### 2.5. In Vitro Biofilm Formation

*T. rubrum* biofilms were formed according to a previously described method [29], with some modifications. *T. rubrum* CBS118892 was grown on malt extract agar for 15 days at 28 °C. The fungal suspension was prepared in 0.9% NaCl, and the conidial concentration was adjusted to approximately 1 × 10^6^ conidia/mL. The plates were initially incubated at 37 °C for 4 h without agitation for pre-adhesion.

### 2.6. Metabolic Activity of the Biofilm by MTT Assay

Following pre-adhesion, treatments consisting of 200 µL each of SRT (12.5 mg/L, and 3.12 mg/L), CASP (15.62 mg/L, and 1.95 mg/L), and SRT + CASP (3.12 mg/L + 1.95 mg/L respectively) prepared in RPMI 1640 medium, supplemented with 2% of glucose, were added into the wells. Biofilms were prepared at different time points (0, 24, 48, 72, and 96 h). Following each incubation, 2 µL of menadione (Sigma-Aldrich) and 20 µL of MTT (Sigma-Aldrich) at 5000 mg/L were added to each well. The plates were incubated at 37 °C for 4 h. Colorimetric changes were measured using an ELISA reader (Thermo Fisher Scientific, Waltham, MA, USA) at 550 nm. A control was prepared by adding 200 µL of untreated conidial suspension to each well.

### 2.7. Quantification of In Vitro Biofilm by Crystal Violet

Following pre-adhesion, the supernatant was removed from the wells and washed thrice with 0.9% sterile saline, after which 200 µL RPMI 1640 medium supplemented with 2% glucose was added to each well. Three independent biological replicates were incubated at 37 °C for 72 h for biofilm maturation. Following biofilm formation, culture medium was removed and treatments comprising 200 µL each of SRT 25 mg/L, SRT 6.25 mg/L, CASP 62.50 mg/L, CASP 1.95 mg/L, and SRT 6.25 mg/L + CASP 1.95 mg/L were added to wells. The treatments were prepared in RPMI 1640 medium supplemented with 2% glucose. Replicates were incubated at 37 °C for 3–7 days. Next, the drugs were removed, and each well was washed thrice with 0.01 M PBS (pH 7.2), following which 100 µL of crystal violet solution was added to each well to quantify biomass. Then, each well was washed twice with sterile water, treated with 100 µL of 95% ethanol and carefully homogenised. The resulting solution was transferred to a new 96-well plate and read using an ELISA reader at a wavelength of 550 nm. A control consisting of 200 µL of untreated conidial suspension was added to each well.

### 2.8. Assessment of Biofilms in Human Nails

The human nail infection assay was performed as previously described [29], with some modifications. Human nail fragments (1 mm^2^) obtained from healthy donors were initially sterilised by autoclaving and transferred to water agar-containing 24-well plates. The fragments were infected with 2 mL suspension prepared in sterile saline 0.9% (NaCl), and conidia concentration was adjusted to approximately 3 × 10^4^ conidia per well. After 4 h of pre-adhesion at 37 °C, the suspension was removed, and each well was washed thrice with 0.9% sterile saline. Next, 2 mL of 0.9% sterile saline was added to each well, following which the plates were incubated for 20 days at 28 °C. Then, 2 mL each of SRT 50 mg/L, SRT 12.5 mg/L, CASP 62.50 mg/L, CASP 3.90 mg/L, and SRT 12.5 mg/L + CASP 3.90 mg/L prepared in 0.9% sterile saline were added to wells daily for seven days. After seven days, the biofilms were analysed using scanning electron microscopy. The assay was conducted according to the Medical School Ethics Committee and approved per protocol number 4.304.317/2020.

### 2.9. Scanning Electron Microscopy

The samples were initially fixed using 3% glutaraldehyde in 0.1% phosphate buffer (*v*/*v*) (pH 7.2) at 4 °C for 24 h and rinsed with 0.1% phosphate buffer (pH 7.2). Osmium tetroxide (1%) was used during the post-fixation step. Subsequently, the samples were dehydrated in an increasing ethanol gradient involving successive baths of increasing ethanol concentrations. Gold was then spray-coated on these samples to visualise biofilms formed in human nails. A JEOL JSM-6610 LV scanning electron microscope at an acceleration voltage of 25 kV was used for visualisation.

### 2.10. Statistical Analysis

We calculated the gene expression using the comparative 2^−∆∆CT^ method. The paired Student’s *t*-test was used to compare gene expression between treatment and control conditions at each time point. The results are reported as the mean ± standard deviation of three independent biological replicas. For comparing the biofilm’s metabolic activity and quantification biomass, one-way ANOVA was used, followed by Tukey’s post hoc test. For all tests, statistical significance was adopted at *p* < 0.05. Prism v. 5.1 (GraphPad Software, San Diego, CA, USA) was used to generate the graphs and statistical analyses.

## 3. Results

### 3.1. Antifungal Susceptibility and Interaction between SRT and CASP

SRT alone and in combination with CASP exhibited antifungal activity against *T. rubrum* free-flowing planktonic cells. The MIC of SRT required to inhibit *T. rubrum* in RPMI medium was 25 mg/L, whereas that of CASP was 31.25 mg/L. The MICs of SRT and CASP increased considerably when the assay was performed in the SB medium. In this medium, SRT showed a MIC of 100 mg/L, whereas CASP showed a MIC of 62.50 mg/L. The interactions between SRT/CASP in both culture media (RPMI or SB) could be considered synergistic (FICI ≤ 0.5). Assays performed in both media indicated that the FICIs corresponding to the SRT/CASP combination ranged between 0.1–0.5. The mean FICIs in the RPMI and SB medium were 0.28 and 0.25, respectively (Table 1). The combined MIC values associated with synergism between SRT and CASP are displayed (Table 2).

### 3.2. RT-qPCR

**Figure 1:**
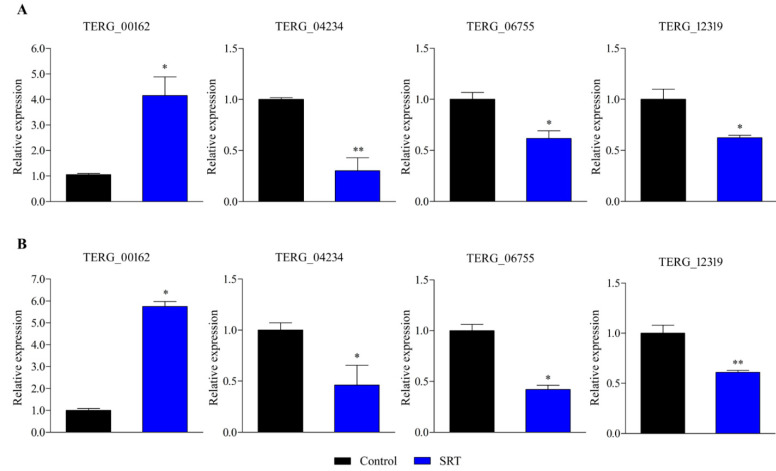
Differentially expressed genes in response to SRT, identified using RT-qPCR analysis. The relative expression of genes *TERG_00162* (MFS multidrug transporter), *TERG_04234* (Hydrophobin), *TERG_06755* (C-8 sterol isomerase), and *TERG_12319* (Chitin synthase 2) at 3 h (**A**) and 12 h (**B**) are represented. Asterisks indicate the statistical significance of the *t*-test compared to the control (SB drug absence). * *p* < 0.05; and ** *p* < 0.01.

RT-qPCR-based gene expression analysis revealed that an SRT concentration amounting to 70% of its MIC induced a gene encoding a transporter (*TERG_00162*) belonging to the major facilitator superfamily (MFS) at 3 and 12 h. In contrast, exposure to SRT for 3 and 12 h downregulated *TERG_12319*, a critical chitin synthase-associated gene linked to fungal virulence and cell wall remodelling. Furthermore, *TERG_04234*, encoding hydrophobin, a putative protein essential for fungal pathogenesis, and *TERG_06755*, encoding a c-8 sterol isomerase protein, required for the ergosterol pathway, were downregulated at 3 and 12 h, respectively (Figure 1).

**Figure 2:**
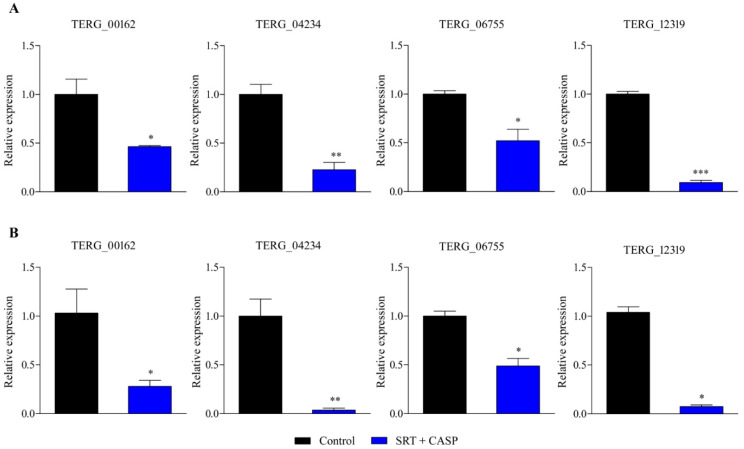
Genes encoding *TERG_00162* (MFS multidrug transporter), *TERG_04234* (Hydrophobin), *TERG_06755* (C-8 sterol isomerase), and *TERG_12319* (Chitin synthase 2) are differentially expressed in response to combinations of SRT with CASP. Asterisks indicate statistical significance determined by *t*-test as compared to the control (SB absence drug) at 3 h (**A**) and 12 h (**B**); * *p* <0.05; ** *p* < 0.01; *** *p* < 0.001.

Gene expression analysis using RT-qPCR was extended to examine *T. rubrum* growth in the presence of the SRT + antifungal CASP combination. SRT + CASP exposure downregulated gene transcription of the MFS multidrug transporter, *TERG_00162*, at 3 and 12 h, in contrast to the induction caused by the SRT alone. In contrast, SRT + CASP downregulated *TERG_04234* and *TERG_06755*, as well as the gene encoding the enzyme chitin synthase, *TERG_12319*, which was similar to that observed under conditions involving the use of SRT alone (Figure 2).

The effects of low concentrations of CASP and SRT on the expression levels of these genes in *T. rubrum* cultured in the presence of CASPsc or SRTsc were analysed. The results showed that SRTsc maintained the expression level of *TERG_00162*, which encodes a multidrug transporter, at a level similar to that observed in the control at 3 h; in contrast, the expression of *TERG_00162* increased at 12 h. Moreover, a significant difference existed between the expression levels shown under these conditions and that of the control (*p* < 0.05). *TERG_06755* expression was downregulated at 3 h, but its expression at 12 h was similar to that of the control. *TERG_04234* and *TERG_12319* maintained their expression under both conditions (control and SRTsc) at 3 h and 12 h (Figure S1).

Exposure of *T. rubrum* to CASPsc resulted in *TERG_00162*, *TERG_04234*, *TERG_06755*, and *TERG_12319* presenting similar expression levels at 3 h or 12 h, compared to that of the untreated control. Therefore, no statistically significant differences existed between these treatment conditions (Figure S2).

### 3.3. Effects of SRT and Its Combination with CASP on the Activity of T. rubrum Biofilm

**Figure 3:**
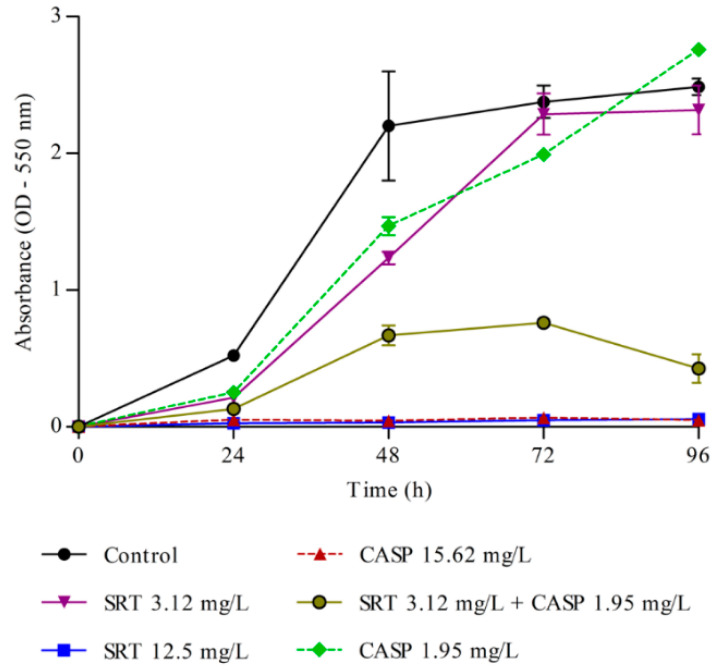
Metabolism of *T. rubrum* biofilm. Effects of sertraline (SRT), caspofungin (CASP), and SRT + CASP on the metabolic activity of *T. rubrum* biofilm.

Metabolic activity in the biofilm was measured every 24 h. A significant increase in metabolic activity was observed up to 72 h, following which the activity stabilised. Therefore, 72 h was considered ideal for biofilm formation. The MTT assay results indicated that 12.5 mg/L SRT alone and of 15.62 mg/L CASP alone, as well as low concentrations of SRT + CASP (3.12 mg/L SRT+ 1.95 mg/L CASP), interfered significantly with the metabolic activities of the biofilm. Treatment with SRTsc or CASPsc did not reduce the metabolic activity of *T. rubrum* biofilms (Figure 3).

**Figure 4:**
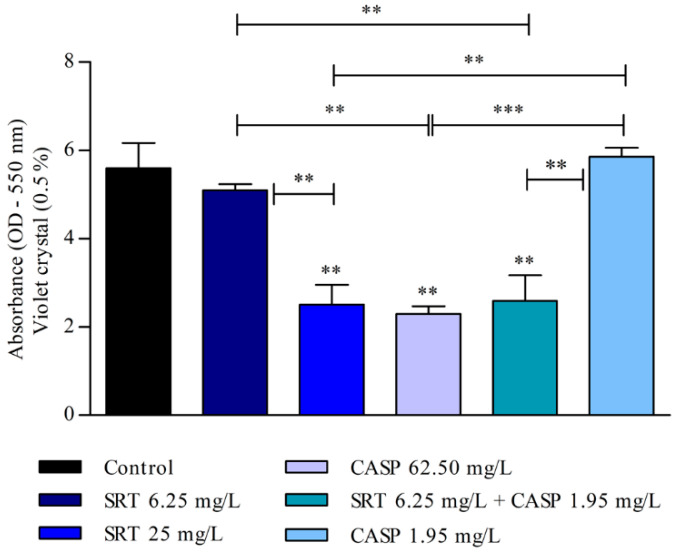
Evaluation of the effect of SRT, CASP, and the synergistic combination (SRT + CASP) on the biomass of *T. rubrum* biofilm stained with violet crystal. The treatments were performed 3 days after the biofilm matured. Statistical analyses showing significant differences (*p* < 0.05) between tested compounds and growth control were estimated using one-way ANOVA and Tukey’s post hoc test; ** *p* < 0.01 and *** *p* < 0.001.

The biomass of the biofilm was also affected by SRT, CASP, and SRT + CASP treatments. Three days after the biofilm was treated with 62.50 mg/L CASP, a significant reduction was observed in the biomass compared with that of the control (*p* < 0.01). Treatment with 25 mg/L SRT as well as 6.25 mg/L SRT + 1.95 mg/L CASP significantly reduced biomass. By contrast, 6.25 mg/L SRT or 1.95 mg/L CASP did not reduce biomass but instead increased production in a manner similar to that of the control (Figure 4).

**Figure 5:**
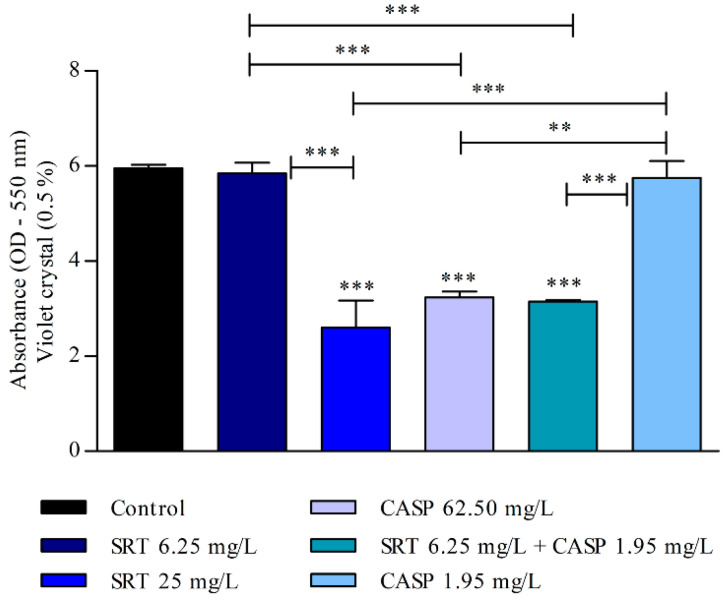
Effect of SRT, CASP, and SRT + CASP on the biomass of *T. rubrum* biofilm. Treatments were performed 7 days after the biofilm matured and stained with violet crystal. Significant differences between tested compounds and growth control were obtained using one-way ANOVA and Tukey’s post hoc test. Asterisks indicate statistical significance: ** *p* < 0.01 and *** *p* < 0.001.

After the biofilm matured for seven days, the 25 mg/L SRT, 62.50 mg/L CASP, or 6.25 mg/L SRT + 1.95 mg/L CASP treatments significantly reduced biomass when compared with the control (*p* < 0.001). However, treatment with 6.25 mg/L SRT or 1.95 mg/L CASP did not reduce the biomass of biofilm that had matured for three days. The biomass produced by these treatments was similar to that observed in the absence of drugs (Figure 5).

The results of the human nail infection assay corroborated the reduction in biomass and metabolic activity of the biofilm, observed via the MTT and crystal violet assays. Scanning electron microscopy revealed that *T. rubrum* forms mature biofilms in human nail fragments after 20 d. Its growth on the substrate was characterised by infinite filaments, which were denser, interconnected, and spread in all directions to form an actual network of connected hyphae. The high antifungal tolerance in *T. rubrum* may be attributed to this network of connected hyphae. Thus, biofilm maturation in human nail fragments was considerably affected by SRT.

**Figure 6:**
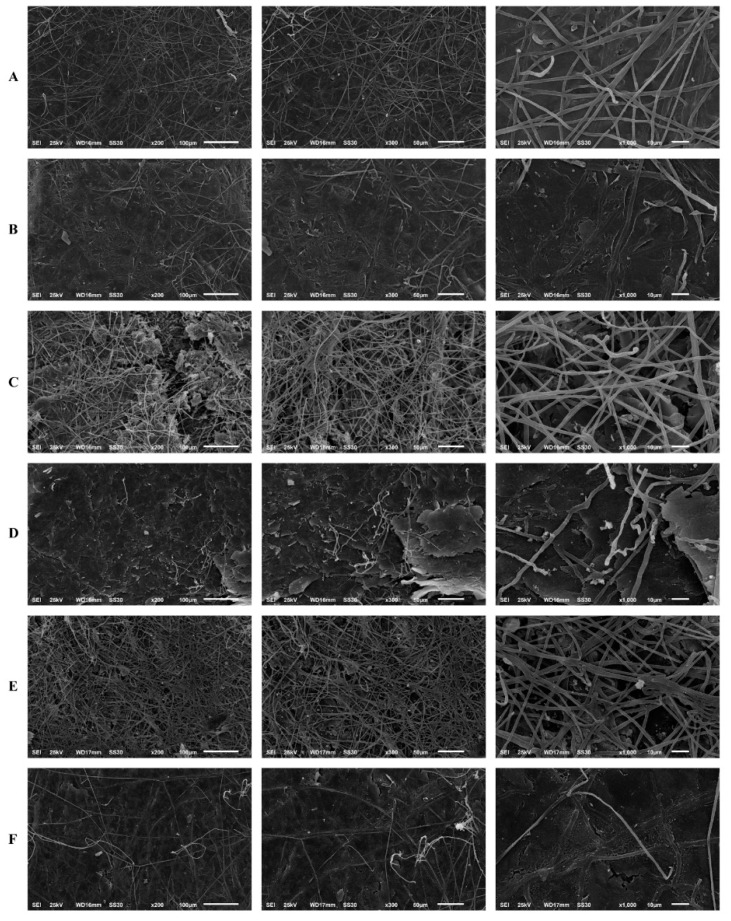
Scanning electron microscopy of the *T. rubrum* biofilm on a human nail. *T. rubrum* strain growth on nail fragments in the absence of drugs (**A**). Treatment with SRT 50 mg/L (**B**), SRT 12.5 mg/L (**C**), CASP 62.50 mg/L (**D**), CASP 3.90 mg/L (**E**) and SRT 12.5 mg/L + CASP 3.90 mg/L (**F**), 20 days of after biofilm matured.

Filament density was significantly reduced by the treatments compared to that in the untreated control. A significant reduction in the thickness of filaments that formed the hyphal network was observed. Additionally, most hyphae could not complete their development to a level that enabled them to create a more uniform biofilm. In this context, the highest activity was observed after *T. rubrum* was treated with SRT and CASP at concentrations corresponding to 2 × MICs, which amounted to 50 mg/L, and 62.50 mg/L, respectively. Excellent biofilm reduction resulting from treatment with 12.5 mg/L SRT + 3.90 mg/L CASP must be highlighted. However, we did not verify the reduction in filament density and the alterations mentioned earlier under isolated, uncombined conditions (Figure 6).

## 4. Discussion

The human antidepressant, SRT, exhibited antifungal activity against planktonic forms of *T. rubrum* (100 mg/L SB and 25 mg/L RPMI media). When combined with CASP, SRT showed excellent synergistic effects by inhibiting *T. rubrum* in vitro. Interaction results showed that the combination of SRT and CASP decreased the concentration of antifungals required to inhibit the fungus by up to 30 times compared to antifungals used individually. These results suggest that repositioning SRT and combining it with CASP constitutes a promising therapy against *T. rubrum*. Several reports have demonstrated SRT activity against pathogenic fungi in vitro and in vivo, with particular reference to *Cryptococcus*, *Candida*, and *Aspergillus* [30,31,32,33]. To the best of our knowledge, we are the first to demonstrate its effect alone and in combination with CASP against the dermatophyte, *T. rubrum*, though a previous study has discussed the advantages of using a combination of SRT and CASP against *Trichosporon asahii* [18]. Moreover, the SRT concentration in the skin is much higher than that in blood; thus, the usefulness of SRT as a treatment against dermatophytosis appears to be promising [34].

We observed that an SRT concentration of 70% of its MIC induced a gene that encodes a transmembrane transporter (*TERG_00162*) belonging to the major facilitator superfamily (MFS). These transporters mediate increased drug efflux, the primary resistance mechanism in dermatophytes [35,36,37]. In this context, the combination of SRT and CASP reduced the transcript levels of the *TERG_00162* gene. Furthermore, exposure of *T. rubrum* to SRTsc or CASPsc (0.273 mg/L SRT or 10.93 mg/L CASP) showed that the expression level of the gene *TERG_00162* was associated with the amount of SRT in the cell and with the activity of the combination.

Fungi may evolve several adaptation mechanisms in response to environmental changes. The fungal cell wall, which enables fungi to interact dynamically with the ambient environment, constitutes a promising antifungal target. Furthermore, its structural integrity, which is actively modulated in response to stress conditions, plays a role in adhesion, signalling, and colonisation, making it essential for the survival of the pathogen [38,39]. Accordingly, other promising antifungal targets were revealed by the treatments, SRT alone and SRT + CASP, which downregulated critical genes that encode chitin synthase (*TERG_12319*) and hydrophobin (*TERG_04234*). However, the expression of these genes did not change when *T. rubrum* was cultured with SRTsc or CASPsc compared to that with the control. This suggested that the efficacy of activity against TERG_04234 and TERG_12319 is due exclusively to the combination of drugs and not to each drug used alone. Chitin, a linear protein that provides strength and protection to many eukaryotes, is vital for cell wall morphogenesis. Chitin is absent in mammalian cells; thus, its metabolism is an attractive target for highly specific antifungal agents [39]. Usually, upregulation of chitin synthesis-related genes is the primary response to cell wall stress [40,41]. The chitin synthase is essential for synthesising chitin in the primary septa of fungi [42]. Hydrophobin, a cysteine-rich protein secreted only by filamentous fungi, lowers the surface tension of water. Identifying drugs that downregulate the genes encoding hydrophobin may help develop novel strategies to treat infections caused by *T. rubrum*. This protein regulates water flux across the fungal cell wall and mediates the attachment of infective structures associated with fungal pathogenesis. Thus, its downregulation is associated with decreased fungal virulence [39,43].

We also identified the effects of SRT and SRT + CASP on the ergosterol pathway. SRT alone at a 70% of its MIC and low concentrations of SRT + CASP downregulated the *TERG_06755* gene, which encodes a c-8 sterol isomerase. This enzyme is involved in the ergosterol biosynthesis cascade [44].The relevance of drugs that target genes or proteins involved in ergosterol biosynthesis has been reported [45].

Our assays demonstrated the impact of SRT and its synergic combination on the metabolism and biomass of *T. rubrum* biofilms. We demonstrated that even low concentrations of SRT, when combined with CASP, are effective against *T. rubrum* biofilms; scanning electron microscopy results substantiated this inhibitory effect. Thus, our results indicate that SRT activity at high concentrations, as well as in combination with CASP, downregulate essential genes related to the formation and constitution of the cell wall, and a gene associated with the ergosterol pathway.

Despite the mechanisms that underlie the effects of CASP on the cell wall of yeasts being known [46], we excluded the possibility that alterations in the expression levels of genes involved in biofilms could be caused by this drug at low concentrations. Our studies indicated that CASP alone or in SC concentrations did not alter the expression of genes involved in cell wall constitution. It did not significantly affect *T. rubrum* biofilms in any of the aspects studied.

Although we used a single reference strain from the *T. rubrum* species, our findings indicate an alternative treatment for dermatophytosis caused by this fungus. SRT combined with CASP minimises fungal resistance and virulence in vitro. However, in vivo studies must also validate our findings and define their clinical utility.

Structural modifications of SRT and CASP molecules would also increase their spectrum of action as antifungal agents, thereby optimising their usefulness against other pathogenic fungi.

## Figures and Tables

**Table 1:** MICs of sertraline (SRT) and caspofungin (CASP) against planktonic forms of *T. rubrum*. FICI and the mean of FICI of interactions between SRT and CASP.

Medium	MIC_100-_mg/L		FICI Range	FICI (Mean)
	SRT	CASP	SRT/CASP	SRT/CASP
SB	100	62.50	0.1–0.50	0.25
RPMI	25	31.25	0.1–0.50	0.28

FICI ≤ 0.5, FICI > 0.5 to ≤4.0, and FICI > 4.0 were categorised as synergism, indifference, and antagonism, respectively.

**Table 2:** MICcb (combined MICs) values for analysis of synergism between sertraline (SRT) and caspofungin (CASP) in assays performed in RPMI medium and Sabouraud (SB) medium.

Medium	Drug	Concentrations Ranged of the SRT (mg/L)								
		200	100	50	25	12.5	6.25	3.12	1.56	0.78
SB	CASP (mg/mL)	0	0	1.95	1.95	1.95	1.95	7.81	15.62	15.62
RPMI		0	0	0	0	0.98	0.98	1.95	1.95	3.90

## Data Availability

The data presented in this study are available in this article and the accompanying Supplementary Data.

## Supplementary Materials

The following supporting information can be downloaded at: https://www.mdpi.com/article/10.3390/jof8080815/s1, Table S1. Primer sets used for real-time quantitative reverse transcription polymerase chain reaction (RT-qPCR). Figure S1. Expression of *TERG_00162* (MFS multidrug transporter), *TERG_04234* (Hydrophobin), *TERG_06755* (C-8 sterol isomerase), and *TERG_12319* (Chitin synthase 2) following SRTsc exposure. Asterisks indicate statistical significance determined by the *t*-test, compared to the control (SB absence drug) at 3 h (A) and 12 h (B); * *p* < 0.05. Figure S2. Differentially expressed genes, *TERG_00162* (MFS multidrug transporter), *TERG_04234* (Hydrophobin), *TERG_06755* (C-8 sterol isomerase), and *TERG_12319* (Chitin synthase 2), following exposure to CASPsc compared to the control (SB absence drug) at 3 h (A) and 12 h (B).
