# Supplementary material for: Synergism between the Antidepressant Sertraline and Caspofungin as an Approach to Minimise the Virulence and Resistance in the Dermatophyte Trichophyton rubrum
Source: J Fungi (Basel). 2022 Aug 3;8(8):815. doi: 10.3390/jof8080815 (PMC9409809; doi:10.3390/jof8080815)
Supplement: Supplementary file 1 [file jof-08-00815-s001.zip › jof-1826942-supplementary.pdf]

Supplementary Data

Synergism between the antidepressant sertraline and caspofungin as an approach to minimise the virulence and resistance in the dermatophyte *Trichophyton rubrum*

Table S1. Primer sets used for real-time quantitative reverse transcription polymerase chain reaction (RT-qPCR).

**Protein/gene**	**ID**	**Primer sequences (5´- 3´)**	**Efficiency (%)**	**Concentration (nM)**
C-8 sterol isomerase	TERG_06755	Fwd: GGTGGGCTTTAGAGTTAG<br>Rev: GCAGTCAAGTAGCAAGTC	110	200
Hydrophobin	TERG_04234	Fwd: GGCATACATCTTGGTGGTTTC<br>Rev: CAGACAGTGGAGGTGGATGTT	103	300
MFS multidrug transporter	TERG_00162	Fwd: CTCCTTTGGACCTTTGATCG<br>Rev: TGACGAAGAGAACGTTGCAG	102	100
Chitin synthase 2	TERG_12319	Fwd: AGCCAACTGCCTTGTACCAT<br>Rev: GTAATCCGACCCATCCCTTT	103.5	200
rpb II	TERG_05742	Fwd: TGCAGGAGCTGGTGGAAGA<br>Rev: GCTGGGAGGTACTGTTTGATCAA	94.99	300
gapdh	TERG_04402	Fwd: GCGTGACCCAGCCAACA<br>Rev: CGGTGGACTCGACGATGTAGT	99.90	200

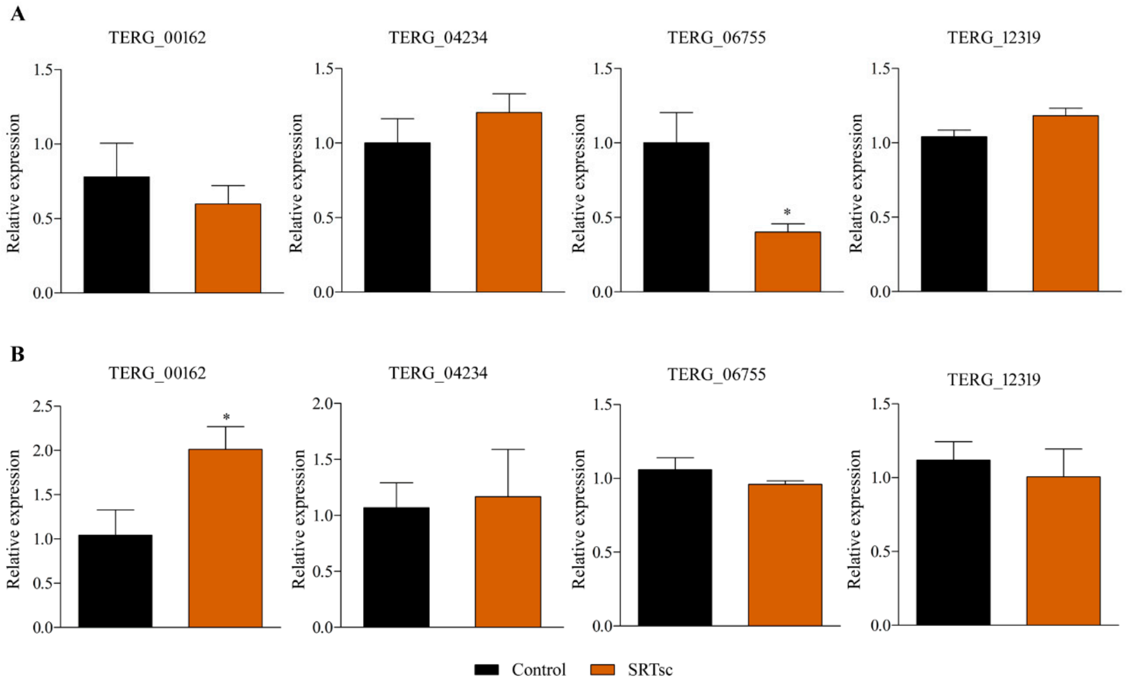

Figure S1. Expression of *TERG_00162* (MFS multidrug transporter), *TERG_04234* (Hydrophobin), *TERG_06755* (C-8 sterol isomerase), and *TERG_12319* (Chitin synthase 2) following SRTsc exposure. Asterisks indicate statistical significance determined by the t-test, compared to the control (SB absence drug) at 3 h (**A**) and 12 h (**B**); *$P < 0.05$.

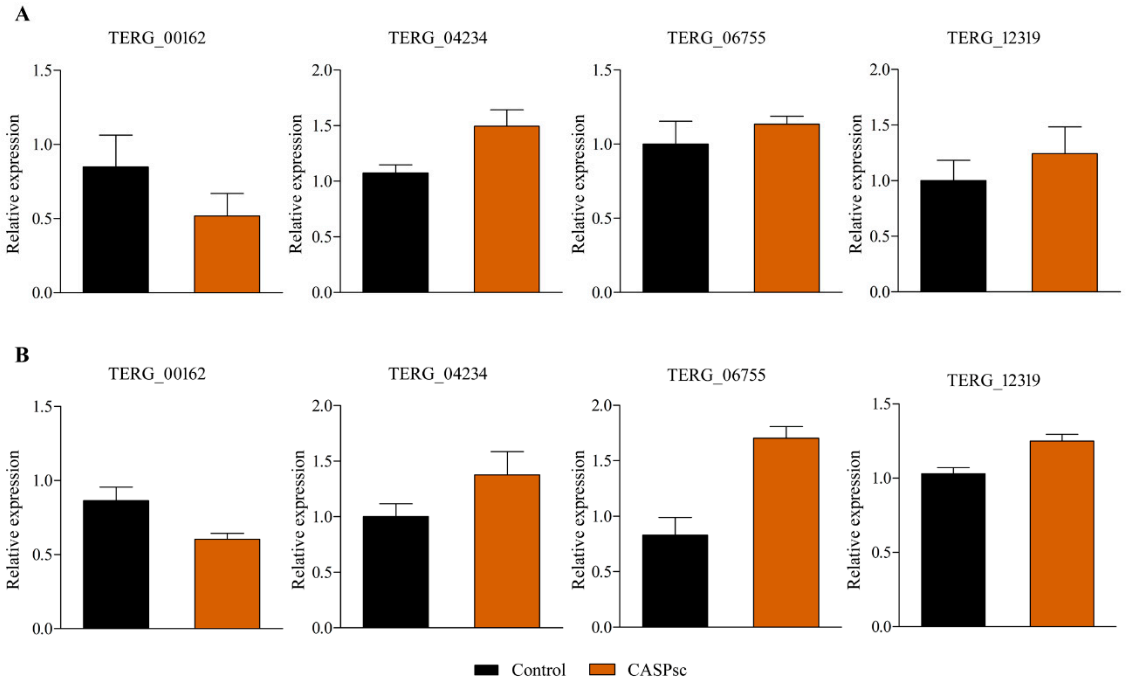

Figure S2. Differentially expressed genes, *TERG_00162* (MFS multidrug transporter), *TERG_04234* (Hydrophobin), *TERG_06755* (C-8 sterol isomerase), and *TERG_12319* (Chitin synthase 2), following exposure to CASPsc when compared to the control (SB absence drug) at 3 h (**A**) and 12 h (**B**).
